# The Relationship of Decision‐making capacity and the age of onset in Alzheimer´s Disease

**DOI:** 10.1002/alz.095399

**Published:** 2025-01-09

**Authors:** Natalie Souza

**Affiliations:** ^1^ IPUB/UFRJ, Rio de Janeiro Brazil

## Abstract

**Background:**

We investigated the relationship of decision‐making capacity in people with young‐onset Alzheimer´s Disease (YOAD) and late‐onset Alzheimer´s Disease (LOAD).

**Method:**

Cross‐sectional study, with 169 (124 people with LOAD and 45 people with YOAD).

**Result:**

People with YOAD (young‐onset Alzheimer´s Disease) were more cognitively impaired when compared with LOAD (late‐onset Alzheimer´s Disease), but more aware of their cognitive deficits and health condition, with moderate effect sizes. All people with AD (Alzheimer´s Disease) presented deficits in the domains of decision‐making capacity, with more impairment in understanding. The majority of participants were in the mild stage of teh disease (CDR 1). The YOAD group had more years of education than the LOAD group.

**Conclusion:**

A higher decision‐making capacity is involved in a better understanding in the YOAD (young‐onset Alzheimer´s Disease) group. Clinically, our findings shed light on the need to consider the differences according to age at onset of AD (Alzheimer´s Disease).

